# Supplementary material for: Surface Properties of CVD-Grown Graphene Transferred by Wet and Dry Transfer Processes
Source: Sensors (Basel). 2022 May 23;22(10):3944. doi: 10.3390/s22103944 (PMC9143786; doi:10.3390/s22103944)
Supplement: Supplementary file 1 [file sensors-22-03944-s001.zip › sensors-1696177-supplementary.pdf]

sensors

Supplementary Materials

Surface Properties of CVD-grown Graphene Transferred by Wet and Dry Transfer Processes

Min-Ah Yoon [1,2,†], Chan Kim [2,†], Jae-Hyun Kim [1,2], Hak-Joo Lee [3] and Kwang-Seop Kim [1,2,*]

[1] Division of Mechanical Engineering, University of Science & Technology (UST), 217 Gajeong-ro, Yuseong-gu, Daejeon 34113, Korea; mayoon@ust.ac.kr (M.-A.Y.); jaehkim@kimm.re.kr (J.-H.K.)
[2] Nano-Convergence Mechanical Systems Research Division, Korea Institute of Machinery & Materials (KIMM), 156 Gajeongbuk-ro, Yuseong-gu, Daejeon 34103, Korea; chankim@kimm.re.kr
[3] Center for Advanced Meta-Materials (CAMM), 156 Gajeongbuk-ro, Yuseong-gu, Daejeon 34103, Korea; hjlee@kimm.re.kr
* Correspondence: kskim@kimm.re.kr
† These authors contributed equally to this work.

1. Roll-based dry transfer of graphene

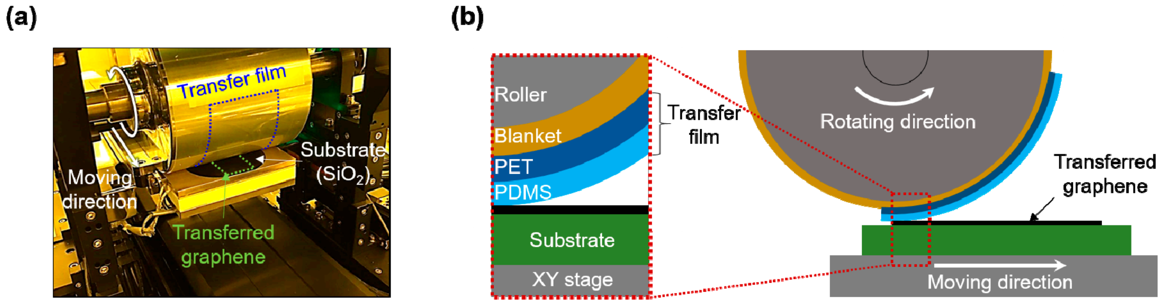

Figure S1. (**a**) A home built roll-to-plate (R2P) transfer machine for the roll-based dry transfer process of graphene. (**b**) Schematic diagram of the side of the TF in contact with graphene in the roll-based dry transfer process.

2. Raman results of wet- and dry-transferred graphene

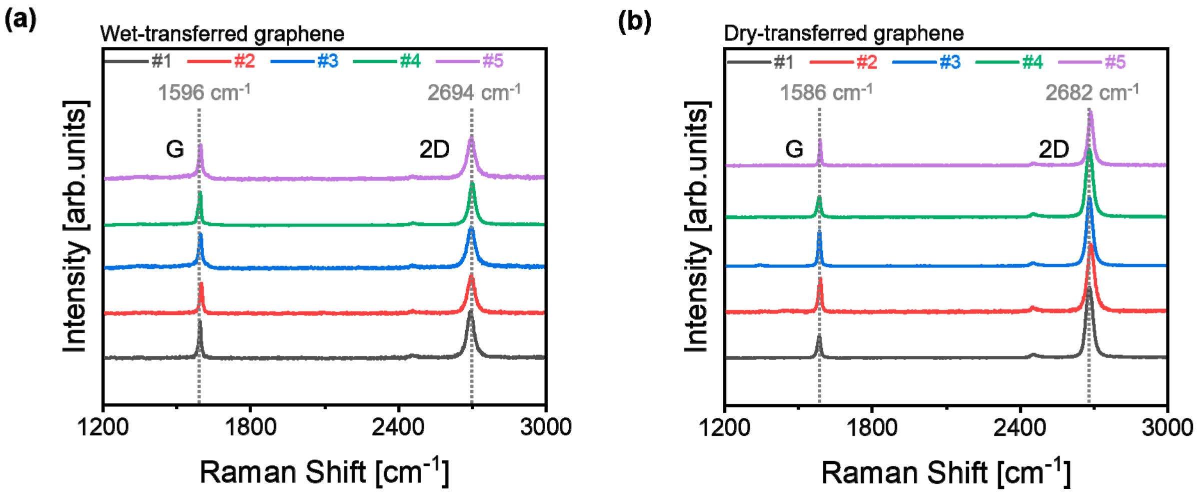

Figure S2. Raman spectra of 5 samples of (**a**) wet- and (**b**) dry-transferred graphene on SiO_2. The dotted line with gray color represents averaged G and 2D peaks of the samples, respectively. The G and 2D peaks of wet-transferred graphene shifted slightly to ω_G ~1,596 cm^{-1} and ω_{2D} ~ 2,694 cm^{-1} from its intrinsic G- and 2D-bands (ω_G ~ 1,580 cm^{-1} and ω_{2D} ~ 2,680 cm^{-1}). Dry-transferred graphene had exhibited similar G and 2D peaks with that of the intrinsic G- and 2D-bands.

Figure S2 shows the Raman spectra of the wet- and dry-transferred graphene on SiO_2. I_{2D}/I_G of the wet-transferred graphene were varied from 1.17 to 1.29 and averaged to 1.22 ± 0.04, which was not significantly changed depending on sample (Fig. S2a). In the dry-transferred graphene (Fig. S2b), I_{2D}/I_G were changed from 1.8 to 3.2 depending on sample and averaged to 2.38 ± 0.6. I_{2D}/I_G of sample #1 and #4 in Fig. S2b were 3 and 3.2, respectively, and it indicates that the samples were slightly doped [1]. However, the difference between I_{2D}/I_G of the wet- and dry-transferred graphene is very clear which means that the transfer methods modified the surface properties of graphene.

References

1. Casiraghi, C.; Pisana, S.; Novoselov, K. S.; Geim, A. K.; Ferrari, A. C. Raman fingerprint of charged impurities in graphene. *Appl. Phys. Lett.* **2007**, 91, 233108.
